# Prevalence of Unprotected Anal Intercourse among Men Who Have Sex with Men in China: An Updated Meta-Analysis

**DOI:** 10.1371/journal.pone.0098366

**Published:** 2014-05-29

**Authors:** Jing Wu, Yifei Hu, Yujiang Jia, Yingying Su, Huixia Cui, Huixin Liu, Ning Wang

**Affiliations:** 1 National Center for AIDS/STD Control and Prevention, China Center for Disease Control and Prevention, Beijing, China; 2 Department of Preventive Medicine, School of Medicine, Vanderbilt University, Nashville, Tennessee, United States of America; 3 College of Nursing, Liaoning Medical University, Jinzhou, Liaoning, China; UNC Project-China, China

## Abstract

**Objective:**

The purpose of this review was to assess the prevalence of unprotected anal intercourse (UAI) among men who have sex with men (MSM) in China.

**Methods:**

A comprehensive search was conducted including online databases like “Wanfang”, Chinese National Knowledge Infrastructure, PubMed and manual searches. Analyses using random-effects models were performed to estimate the prevalence of UAI among MSM in China.

**Results:**

Sixty-two articles reporting eighty-two studies were selected. The pooled prevalence rates of UAI with any male partner, with regular male partners, with non-regular male partners, with casual male partners, and with commercial male partners among MSM were 53%(95%CI: 51–56%), 45%(95%CI: 39–51%), 34%(95%CI: 24–45%), 33%(95%CI: 30–36%), 12% (95%CI: 5–26%), respectively. A cumulative meta-analysis found that the pooled UAI prevalence decreased over time.

**Conclusions:**

Although the prevalence of UAI with male partners among MSM in China presents a decreasing trend over the past decade, the concomitant rise in HIV prevalence and incidence indicates that current prevention intervention efforts are insufficient to effectively contain the spread of HIV. Therefore, the persistently high prevalence of risky sexual behaviors underscores the need for innovative and effective prevention strategies among MSM.

## Introduction

Approximately 7.3%, 11.0%, 14.7% and 17.4% of the estimated number of people living with HIV/AIDS in China were infected through unprotected male-to-male sexual contact in 2005, 2007, 2009 and 2011, respectively, reflecting a rapid expansion of the HIV epidemic among men who have sex with men (MSM) in recent years [1]–[4]. Data from meta-analyses [5]–[8] and sentinel surveillance [8], [9] have consistently demonstrated the rising trend of HIV prevalence and incidence among MSM across the nation. Estimated HIV prevalence and incidence increased to 6.0% (4.4–8.2) and 1.0 (0.7–1.3) per 100 person-years, respectively, in 2010 [8], which highlights the magnitude of the HIV epidemic in this population. Recent studies have consistently revealed the rapid expansion and severity of the epidemics of HIV and other sexually transmitted infections (STIs) among MSM in large metropolitan cities in China [6], [10]–[15].

The disproportionately high prevalence of HIV and STIs among MSM is associated with the prevalence of unprotected anal intercourse (UAI) [16], [17], which has been the leading high-risk behavior for HIV infection [18]–[21]. HIV can spread rapidly in a network with a high proportion of HIV-positive male partners engaging in UAI, or with seminal fluid ejaculated inside the rectum [22]–[24]. Men who report receptive UAI (URAI) are at higher risk of HIV infection, compared to men who report insertive UAI (UIAI) [20], [22], [23]; the risk increased by more than three-fold in an Australian cohort of homosexual men [23]. In addition, anal sex is more likely to be unprotected with regular male partners than with casual or commercial male partners [25]–[28], which is challenging for encouraging behavioral changes between steady male couples because UAI implies a commitment of love, fidelity and intimacy according to their perceptions [29], [30]. It cannot be denied that HIV-negative men in discordant couples have the highest risk of acquiring HIV among MSM [22], [23]. Moreover, increasing UAI may also offset the effects of antiretroviral therapy, even lead to HIV resurgence [32], [34], [35]. Conversely, empirical data and mathematical models have shown that decreasing risk behaviors are accompanied by decreasing reproduction numbers [31], [32]. Here the reproduction number is defined as the average number of secondary cases caused by a typical HIV case during its infectious period [33].

An earlier meta-analysis [36] reported that a decreasing trend was observed at last UAI with commercial partners, but no significant change at last UAI appeared prior to the year 2007 among MSM in China. The Chinese government realized the alarmingly high risk among MSM and scaled up intervention efforts among this group [37], and the first nationwide cross-sectional survey among MSM in 61 large cities was initiated in 2008 [38]. It is imperative to evaluate the prevalence of UAI among MSM in order to further guide intervention and prevention programs. This article systematically reviewed published studies to estimate UAI prevalence and characterize its trend among MSM in China.

## Methods

This systematic review followed the guidelines of preferred reporting items for systematic reviews and meta-analyses published in PLoS Medicine (Checklist S1) [39].

### Search strategy

Searched online databases included “Wanfang” (including more than 5700 Chinese journals issued since 1998), Chinese National Knowledge Infrastructure (CNKI, including more than 7500 Chinese journals issued since 1994), and PubMed up to September 2013. Searches of “Wanfang” and CNKI as Chinese databases focused on published articles. Searches were filtered by titles, abstracts and keywords in the two Chinese databases: (“gang jiao” or “gang men xing jiao” or “gang men xing xing wei”, meaning “anal sex”) and (“nan tong” or “tong zhi” or “nan nan xing xing wei” or “nan nan xing jie chu” or “tong xing lian” or “MSM”, meaning “male-to-male sex act”). The following terms combined were searched by all fields or by MeSH terms in PubMed database: (“anal sex” or “anal intercourse” or “condom use” or “risk behavior”) and (“homosexual” or “bisexual” or “gay” or “MSM” or “men who have sex with men” or “tongzhi”) and “China”. The relevant published articles were examined by hand for additional studies, but none fulfilled the study selection criteria.

### Study selection

In this meta-analysis, UAI was defined as occurring with male partners and included both UIAI and URAI. Studies were included if they met all of the following criteria: conducted in mainland China; reported the prevalence of UAI or UIAI or URAI with male partners in the last six months; and reported the location of the study (names of provinces were provided), data collection time, and sample size. UAI information included the following: UAI or UIAI or URAI with male partners (e.g., any [of the following three types], regular, commercial, and casual; or non-regular including commercial and casual). Additional rules (see below) were applied to help data extraction. If multiple publications covered the same study, the most comprehensive article or the article providing more UAI information or the earliest published article (in order of decreasing priority) was selected. We contacted some authors to verify possible multiple publications of the same study. Only baseline information from cohort studies was used in this study. All UAI data were extracted from a given study if information on UAI prevalence by subgroups was available. Authors were contacted to clarify any questions related to UAI data, study design, and data representativeness.

### Data extraction

The information from selected articles was extracted and entered into a Microsoft Excel 2010™ (Microsoft Corporation, Redmond, Washington, USA) by one reviewer and reviewed separately by another independent reviewer according to the selection criteria. A third independent reviewer reconciled the discrepancies. We extracted the following variables: first author, year of publication, data collection period, study location (including cities and provinces), publication language, sampling method, age (mean ± SD and/or median, range), sample source, study type, data collection method, HIV prevalence, recruitment settings (defined as the facilities where data were collected, e.g., entertainment venues, offices of Centers for Disease Control), UAI type, sample size, and the number of UAI events among MSM.

### Quality Assessment

Quality assessment of the included studies was conducted based on EBMH guidelines for evaluating prevalence studies [40]. Eight items were scored as either 1 or 0, which corresponded to “yes” or “no”, and the total scores ranged from 0 to 8 (Checklist S2).

### Statistical methods

All analyses were conducted using a Meta package of R software version 3.0.1 and 3.0.2 (R Foundation for Statistical Computing, Vienna, Austria). Logit transformation was applied to reduce the variances among UAI prevalence of MSM in individual studies. The inverse variance methods and random-effects models were used to determine the weight of each study. Pooled UAI prevalence with any male partner, with regular male partners, with non-regular male partners, with casual male partners, and with commercial male partners, as well as their 95% confidence intervals (95%CI), were reported. A cumulative meta-analysis was performed to trace the dynamics of annual UAI prevalence [41]. Sub-group analyses were conducted to find any differences between stratified variables, e.g., data collection period, region, sampling method, study type, data collection method, recruitment settings and HIV prevalence. Variables with a P-value ≤0.05 in sub-group analyses entered multivariate meta-regression analysis together, and a mixed-effects model was used. Heterogeneity was tested by Q statistics and quantified by I-square statistics. Publication bias was examined by a linear regression test to assess if funnel plots were symmetric (the threshold of P value is 0.05). In the sensitivity analyses, we compared the estimated UAI prevalence among MSM of k studies and UAI prevalence of k-1 studies to determine the robustness of the pooled prevalence. Here, k is the number of studies included in the meta-analyses.

## Results

### Characteristics of studies

**Figure 1:**
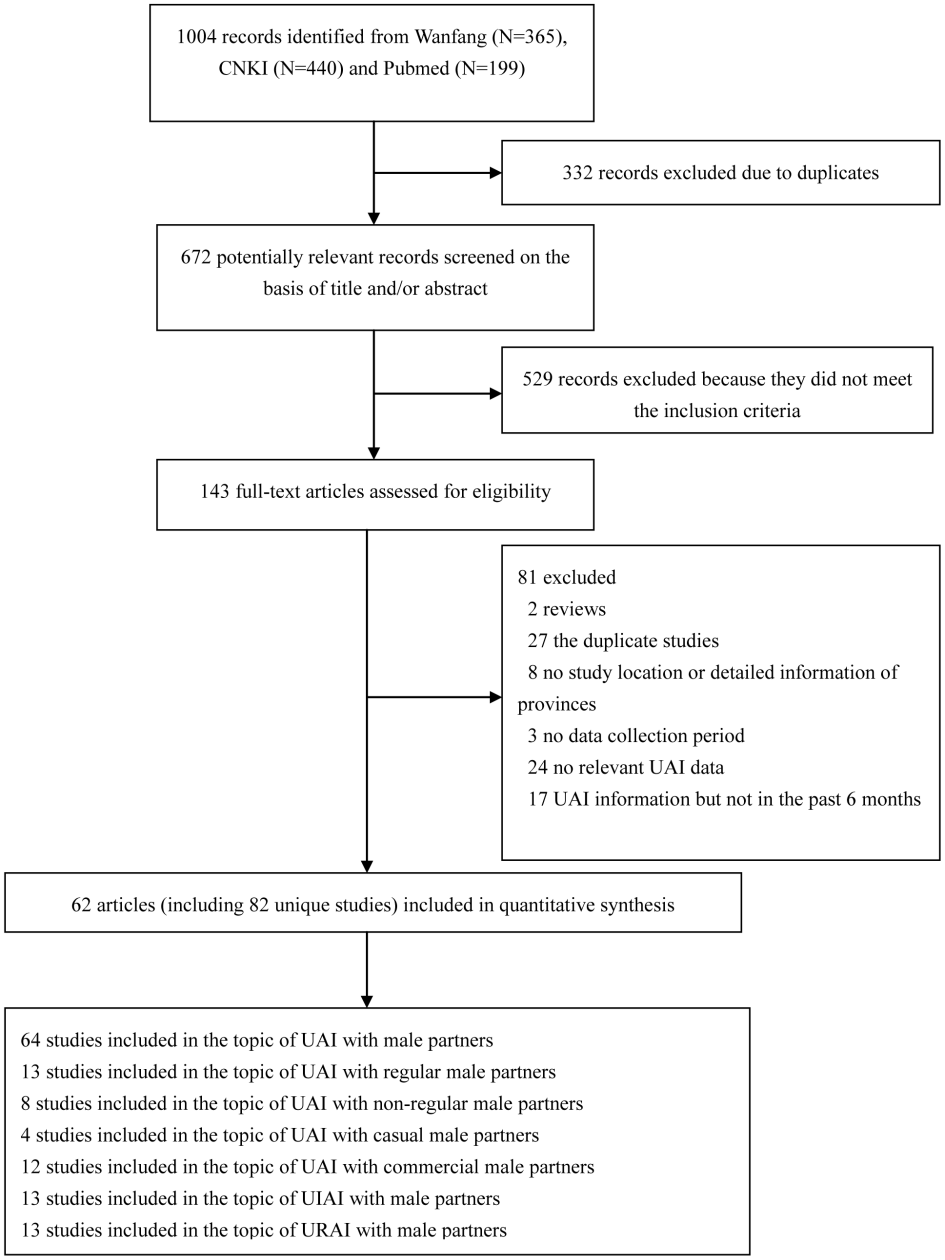
Flow Diagram of the Study Identification and Selection.

A total of 82 unique studies from 62 publications were selected and included in this meta-analysis (Figure 1). One [42] of the 62 selected publications presented five cross-sectional studies in different years, two publications presented four cross-sectional studies each (one in different cities [43], and one in different years [44]), three publications included three cross-sectional studies each in different years [45]–[47], and four publications included two cross-sectional studies each (one in different HIV infection status [48], two in different cities each [49], [50], and one in different years [51]). Each remaining publication presented one individual study. Table S1 presents a summary description of the 82 studies [42]–[103].

A total of 41203 MSM provided information on UAI in the 82 studies (31 studies in English and 51 in Chinese). Among the 82 studies, 23 were conducted from 2001 to 2007, 56 from 2008 to 2012, and another three across from 2007 to 2008 [49], [95]. The participants were recruited from MSM venue-based methods, either in conjunction with or exclusive of other methods, in about half of the studies (42/82). Sampling methods adopted included respondent driven sampling (RDS), snowball sampling, time-location-sampling method, and convenient sampling. The majority of the studies used cross-sectional study designs (72/82) and face-to-face interviews (58/82). These included 82 studies were conducted across 18 of 31 provinces, municipalities, and autonomous regions in China, and nearly three-quarters of the studies were conducted in Beijing, Chongqing, Heilongjiang, Jiangsu and Sichuan. About 80% (27/34) studies collected data in medical facilities among the 34 studies providing information on recruitment settings, while 48 studies failed to provide such information. The ages of participants in 38 studies ranged from 15 to 78 years, and the median mean age of 53 studies was 28.0 years (range: 20.2–36.3). The median HIV prevalence of 64 studies was 6.2% with a range of 0 to 31.6%. The scores of quality assessment ranged from 2 to 6, with a median of 4 in all included studies, and the results were similar to another meta-analysis [104]. The median scores for each item were 1, 0, 1, 0, 1, 0, 1, 0, respectively.

### UAI prevalence estimates with male partners

**Figure 2:**
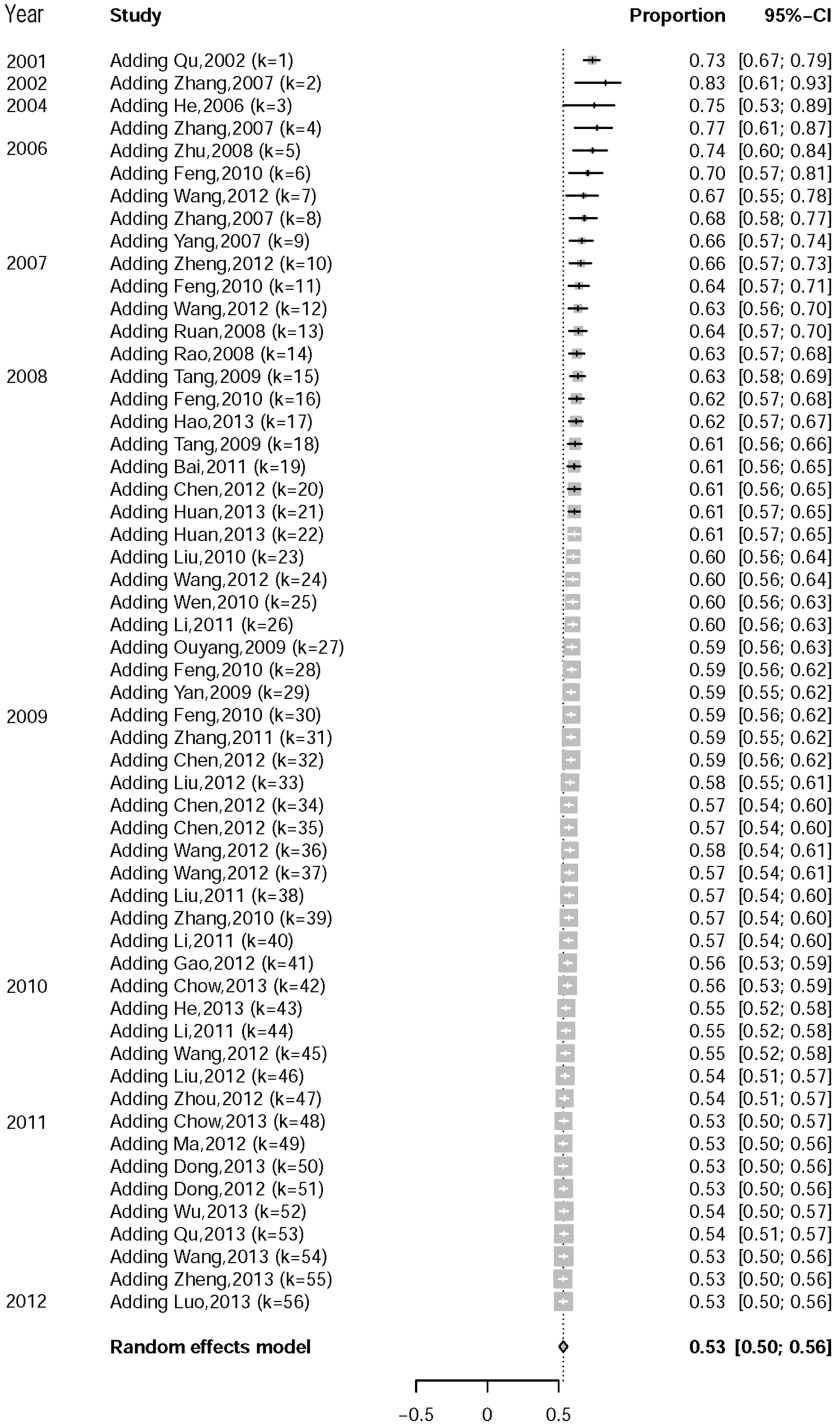
Cumulative Meta-analysis of UAI with Male Partners among MSM in China.

Table 1 and Figures S1-S7 present the UAI prevalence with different types of sexual partners. The pooled UAI prevalence with any male partner (53%, 95%CI: 51–56%) was statistically higher than the UAI prevalence with non-regular (34%, 95%CI: 24–45%), casual (33%, 95%CI: 30–36%), or commercial (12%, 95%CI: 5–26%) male partners, and also higher than the prevalence of UIAI or URAI with any male partner (UIAI: 43%, 95%CI: 36–49%; URAI: 38%, 95%CI: 33–44%). The rates of UAI with different types of male partners ranked in the following descending order: regular male partners (45%, 95%CI: 39–51%), casual male partners, and commercial male partners. A cumulative meta-analysis showed a decreasing trend in prevalence over time (Figure 2). High heterogeneity was found in the overall UAI prevalence with any male partner (Q = 1,308, P<0.0001), with regular male partners (Q = 189, P<0.0001), with non-regular male partners (Q = 198, P<0.0001), with commercial male partners (Q = 634, P<0.0001), UIAI or URAI with any male partner (Q = 269, P<0.0001; Q = 243, P<0.0001). However, no obvious publication bias was observed in all of the above UAI prevalence rates (Table 1). Sensitivity analyses showed no obvious difference between k outcomes from k-1 studies and the outcome from k studies.

**Table 1:** The Pooled Prevalence of UAI with Male Partners.

UAI type	No. of studies	Pooled sample size	Number of MSM with UAI	M (IQR) (%)	Pooled UAI prevalence and 95%CI (%)	P value of publication bias
UAI with any male partner	64	31362	16263	53(13)	53(51–56)	0.09
UAI with regular male partners	13	4007	1665	43(10)	45(39–51)	0.12
UAI with non-regular male partners	8	2632	724	33(25)	34(24–45)	0.16
UAI with casual male partners	4	1132	373	32(2)	33(30–36)	0.47
UAI with commercial male partners	12	4340	389	5(40)	12(5–26)	0.55
UIAI with any male partner	13	5763	2383	43(13)	43(36–49)	0.31
URAI with any male partner	13	5763	2154	36(12)	38(33–44)	0.45

Note: M: median; IQR: inter quartile range.

### Sub-group and meta-regression analyses to partially clarify the heterogeneity of UAI prevalence with any male partner

The sub-group analyses (Table 2) showed significant factors for the heterogeneity of UAI prevalence were data collection period, sampling method and HIV prevalence. However, the above variables explained only about 20% of the variance (R^2^ = 0.20) from the UAI prevalence of individual studies in multivariate meta-regression analysis (Table 3). The variable of data collection period significantly influenced UAI prevalence with any male partner.

**Table 2:** The Pooled UAI Prevalence with Any Male Partner, by Subgroups.

Stratified variables	No. of studies	Pooled sample size	Number of MSM with UAI	Pooled UAI prevalence and 95%CI (%)	Q_B_	P value
Data collection period					15.74	0
2001–2007	17	7909	4708	61(57–66)		
2008–2012	47	23453	11555	50(48–53)		
Region†					1.18	0.88
Northeast	12	6217	3152	57(49–65)		
North China	5	3003	1592	55(47–62)		
East China	23	9567	4982	53(49–57)		
Southwest	15	7971	4365	53(48–57)		
South central	7	3604	1685	51(42–60)		
Sampling method					8.68	0
RDS	10	3593	2166	61(56–67)		
Other methods	54	27769	14097	52(49–55)		
Study type					3.17	0.08
Cross-sectional study	56	29053	14915	52(50–55)		
Other types	8	2309	1348	60(52–68)		
Data collection method					0.77	0.68
Interviewer	50	25997	13496	53(50–56)		
Self	5	2418	1194	51(45–57)		
both	3	1157	565	50(43–57)		
HIV prevalence					5.8	0.02
≤5%	24	11776	6058	57(51–61)		
>5%	27	15898	8129	49(46–52)		
Recruitment settings					3.99	0.14
Medical facilities	24	9764	4939	52(49–55)		
MSM venues	6	2909	1657	60(53–67)		
No report	34	18689	9667	53(49–57)		

† : Region division referred the government website (http://www.xzqh.org.cn/ONEWS_zq.asp?id=6162). Two articles were excluded: one in Ningxia in the northwest region, and one in Guangdong and Sichuan across two regions.

**Table 3:** Multivariate Meta-regression Analysis of UAI Prevalence with Any Male Partner.

Variables	β(95% CI)	Standard error	Z	P value
Data collection period (reference = 2008–2012)	0.51(0.26–0.76)	0.13	4.02	0
Sampling method (reference = non-RDS)	0.31(−0.04–0.65)	0.18	1.74	0.08
HIV prevalence (reference>5%)	−0.09(−0.32–0.14)	0.12	−0.75	0.45

## Discussion

Our meta-analysis estimated UAI prevalence with different types of sexual partners and sexual positions among MSM (including both HIV infected and non-infected individuals) in China. The findings of UAI prevalence from this meta-analysis are robust based on the stable outcomes from the sensitivity analyses. The estimated UAI prevalence with any male partner among MSM in China was 53% (95%CI: 51–56%), which was significantly lower than that (75%; 95%CI: 67–82%) estimated among HIV-infected MSM in a China meta-analysis [105]. The pooled UAI prevalence with any male partner among MSM was 55% (95%CI: 52–58%) in 2008 and 50% (95%CI: 44–57%) in 2009 (data were not shown), which was similar to the 52% reported in the unprecedented nationwide survey targeting MSM in 61 metropolitan cities of China in 2008–2009 [38]. Furthermore, UAI prevalence with any male partner among MSM in China was comparable with that among MSM from the National HIV Behavioral Surveillance System (NHBS) across 21 U.S. cities in 2008 (54%) [25]. Studies [18], [106], [107] evidenced that UAI is an independent predictor associated with HIV and other STIs. The findings of this study reveal the considerable number of MSM practicing UAI with their male partners, which could be a major driving force for the rapid spread of HIV and other STIs among MSM in China.

This study showed a statistically decreasing trend in prevalence rates of UAI with regular male partners, with casual male partners and with commercial male partners. A similar decreasing trend was observed in other studies as well as in the two cycles' surveys of the NHBS System in U.S. [25]–[28]. These all showed that MSM decide upon risky or safe behaviors according to their perception of intimacy levels with male partners. The estimated UAI prevalence rates with regular male partners and with non-regular male partners (43% and 34%) were higher than those reported in NHBS in U.S. (37% and 25%) [25]. Condom use with a regular male partner is a complex topic because it may carry implications about mutual trust and stability in the monogamous relationship [29], [30]. Some factors such as the duration of the monogamous relationship and unprotected sex acts outside of the relationship may increase the potential transmission and spread of HIV and other STIs [30], [108]. The high risk of UAI with regular male partners contributes much to HIV epidemic [29], so encouraging positive social norms about condom use in MSM's mainstream contexts may have a positive intervention impact. This study found that 12% of MSM engaged in UAI with commercial male partners, which might be cautiously explained by the prevalence of sexual dysfunction; the prevalence of erectile dysfunction among MSM in China was about 10% [109]. Additionally, we need to investigate further the syndemic risk factors of the high UAI prevalence with casual male partners from individual, interpersonal and socio-cultural perspectives [110] so that interventions can be prioritized and tailored to the specific subgroups of MSM.

URAI is recognized as carrying a higher risk of acquiring HIV than UIAI [20], [22], [23], which is sometimes referred to as a “strategic position.” It is cautious to apply this strategy to control HIV epidemics despite its lower risk than URAI, since MSM with UIAI are proven to have a higher risk of HIV transmission than MSM without UAI [23]. HIV-negative MSM should protect themselves from HIV by adopting safer sex behaviors, rather than relying on the less risky but still dangerous strategy of UIAI.

A declining UAI prevalence with male partners among MSM in China over time was observed in this study, but the epidemics of HIV and other STIs remained rapidly expanding [5]–[8]. An inverse correlation between UAI prevalence and HIV prevalence has been also reported in five consecutive cross-sectional studies in Harbin, a provincial capital of Heilongjiang, China [42]. However, a previous Chinese meta-analysis [36] did not find the inverse correlation between UAI prevalence and HIV/syphilis prevalence. Our meta-analysis and other HIV/STIs meta-analyses [5]–[8] among MSM in China retrieved and included more studies than the previous meta-analysis [36]. Our meta-analysis included studies conducted over a longer interval from 2001 to 2012, similar to a U.S. meta-analysis [24]. However, the previous meta-analysis [36] showed that the included studies were conducted from 2003 to 2007. Our meta-analysis included studies beyond 2008, which was a critical year when more attention began to be paid to MSM [37], [38]. In addition, we performed more thorough analyses to understand UAI characteristics in China, such as cumulative meta-analysis, meta-regression, and subgroup analyses. In conclusion, the outcomes from our meta-analysis may be more convincing.

The inverse relationship between UAI prevalence and HIV/STIs prevalence may be attributed to several reasons. Outcomes from mathematical model studies [111]–[114] suggest that it requires more than 80 percent coverage in condom use to curb HIV epidemic. HIV transmission probability is a sensitive factor influencing HIV epidemic [115], [116], and higher transmission risk through anal rather than vaginal sex [117], [118] also intensifies HIV transmission among MSM. In addition, high HIV prevalence may be attributed to unprotected group sex [76], [119]; the prevalence of group sex among MSM in China ranged from 11% to 38% [100], [120]. Obviously a higher prevalence of group sex predicts a higher risk of UAI among MSM [121]. The decreasing UAI prevalence over time shows that intervention efforts among MSM in China are effective to some degree. However, the highly prevalent rate of UAI concurrent with a high prevalence of HIV-infected partners in sexual networks fuels the rapid spread of HIV. Therefore, China should develop more effective and better targeted interventions among MSM in a sustainable way to contain HIV spreading. Decreasing UAI exposure is an important goal that can be addressed by providing condoms, behavior change and communication.

Only 20% of the variance from UAI prevalence of individual studies can be explained by data collection period, sampling method and HIV prevalence in this meta-analysis, and more variables are needed to explain the heterogeneity. Though the study did not find a difference in UAI across different regions, UAI prevalence showed high diversity across cities (Q = 12, P = 0.03, Figure S8). HIV status consistent with UAI is also an important factor in explaining the heterogeneity of UAI prevalence among MSM. Future epidemiologic surveys should address the in-depth correlations between interventions and HIV prevalence of MSM and provide intermediate evidence of HIV spread in China. The high heterogeneity across the individual studies incorporated in this meta-analysis was not unique; it was also found in other meta-analyses of MSM in China [5]–[7], [36], [105]. High heterogeneity may result from variations in the study locations, data collection time and sampling methods. Prior China- and U.S.-based UAI meta-analyses showed similar high heterogeneity (P<0.0001) [24], [36], [105]. It seems that heterogeneity is unavoidable in MSM studies based on non-probability sampling and participants' recruitment strategies.

We recognized the limitations of this meta-analysis. First, the research studies were usually reported and conducted in severely endemic areas, which could lead to a potential overestimation of the prevalence of UAI. Second, this study did not differentiate between the rates of UAI among HIV infected and non-infected groups. Serosorting and strategic positioning [23], [30], [122]–[124] were also not explored in this study.

In summary, the findings of this meta-analysis revealed a high prevalence of UAI with male partners and a decreasing trend among MSM in China. The declining rates of UAI among MSM in China suggest that preventive intervention efforts in recent years may slow the increasing rate in HIV prevalence and incidence, but are not enough to control HIV spreading. These interventions include condom promotion, peer education, media-based education and counseling, and HIV testing and counseling [125], [126], which are all independently associated with lower UAI prevalence [97]. In addition, the following factors may lead to a higher risk of UAI: a reluctance to use condoms, multiple partners, and alcohol and/or substance use before sex [107]. However, the continuously high rate of UAI underscores the need for innovative and effective prevention intervention strategies among MSM in China.

## Supporting Information

Figure S1 **Forest plot of UAI prevalence with any male partner among MSM in China.**
(TIF)

Figure S2 **Forest plot of UAI prevalence with regular male partners among MSM in China.**
(TIF)

Figure S3 **Forest plot of UAI prevalence with non-regular male partners among MSM in China.**
(TIF)

Figure S4 **Forest plot of UAI prevalence with casual male partners among MSM in China.**
(TIF)

Figure S5 **Forest plot of UAI prevalence with commercial male partners among MSM in China.**
(TIF)

Figure S6 **Forest plot of UIAI prevalence with any male partner among MSM in China.**
(TIF)

Figure S7 **Forest plot of URAI prevalence with any male partner among MSM in China.**
(TIF)

Figure S8 **Subgroups analysis of UAI prevalence with any male partner among MSM in different cities of China.**
(TIF)

Checklist S1 **PRISMA Checklist.**
(DOC)

Checklist S2 **Quality assessment of included individual studies.**
(DOC)

Table S1 **Characteristics of studies included in this meta-analysis.**
(DOCX)
