# Supplementary figures and images for: Prevalence of Unprotected Anal Intercourse among Men Who Have Sex with Men in China: An Updated Meta-Analysis
Source: PLoS One. 2014 May 29;9(5):e98366. doi: 10.1371/journal.pone.0098366 (PMC4038612; doi:10.1371/journal.pone.0098366)

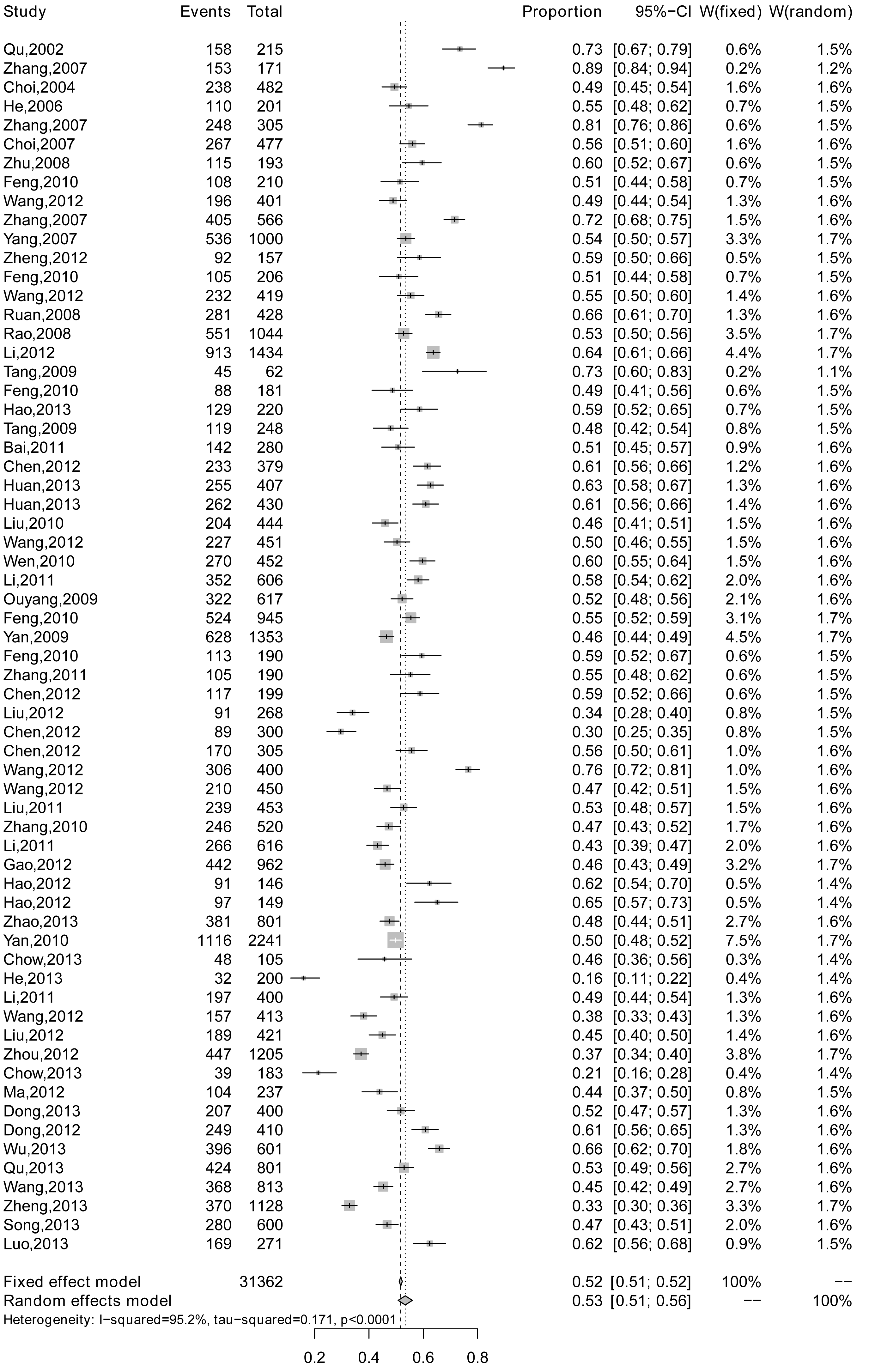

Supplement: Figure S1 — Forest plot of UAI prevalence with any male partner among MSM in China. (TIF) [file pone.0098366.s001.tif]

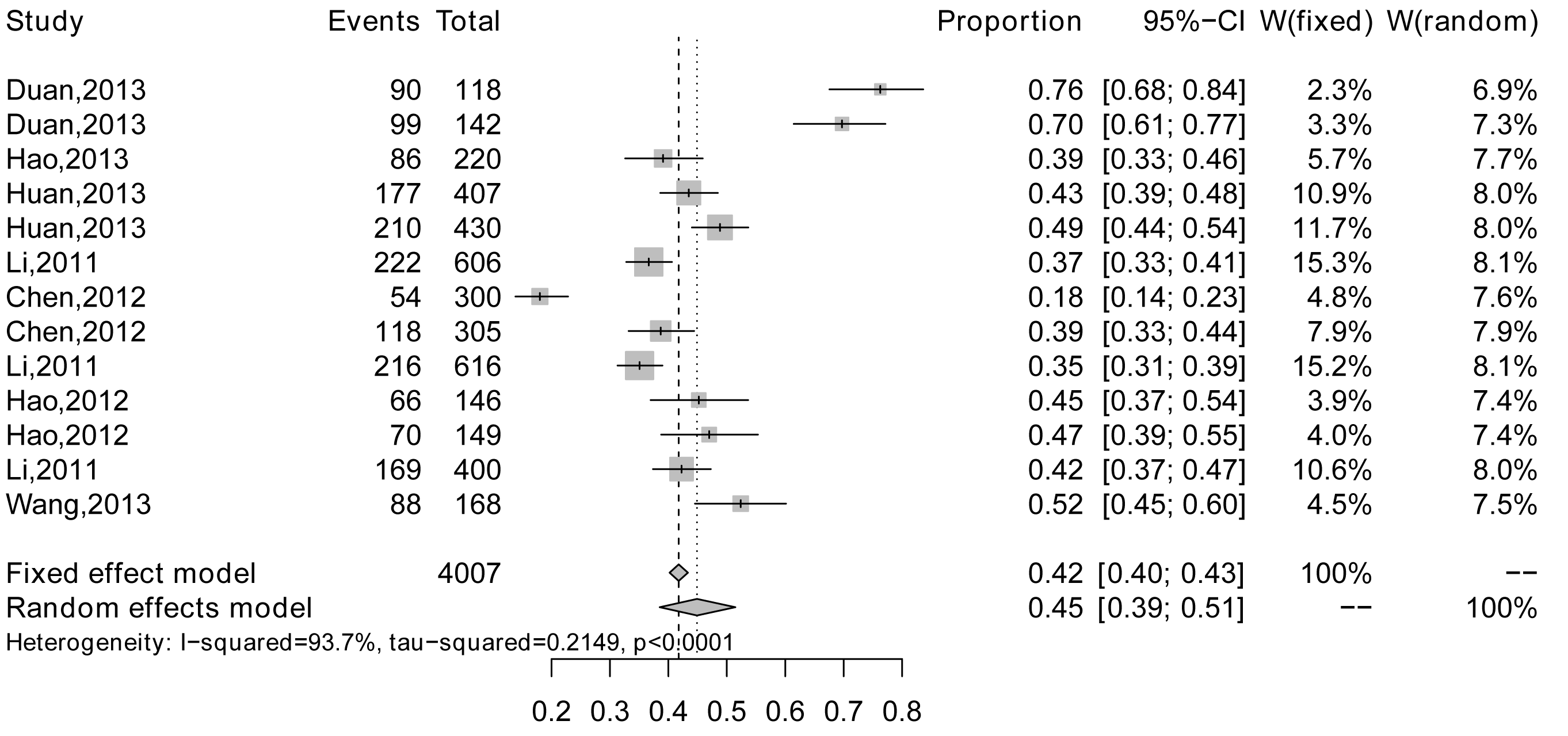

Supplement: Figure S2 — Forest plot of UAI prevalence with regular male partners among MSM in China. (TIF) [file pone.0098366.s002.tif]

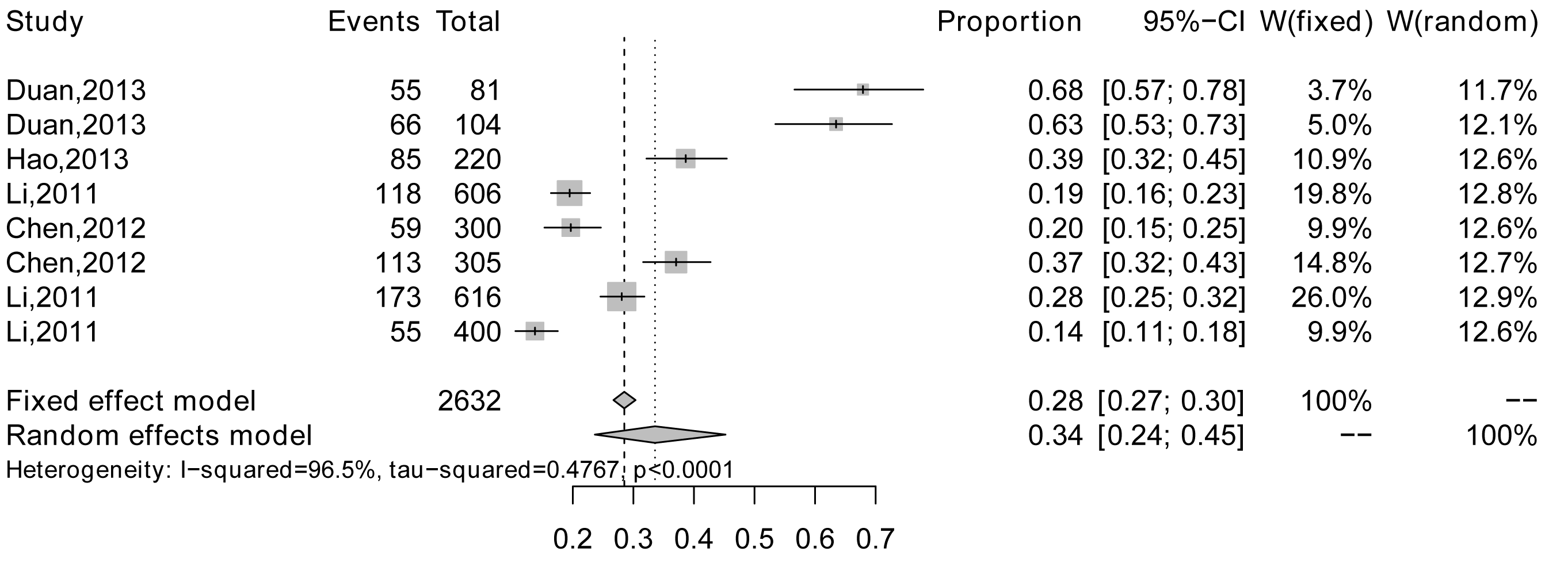

Supplement: Figure S3 — Forest plot of UAI prevalence with non-regular male partners among MSM in China. (TIF) [file pone.0098366.s003.tif]

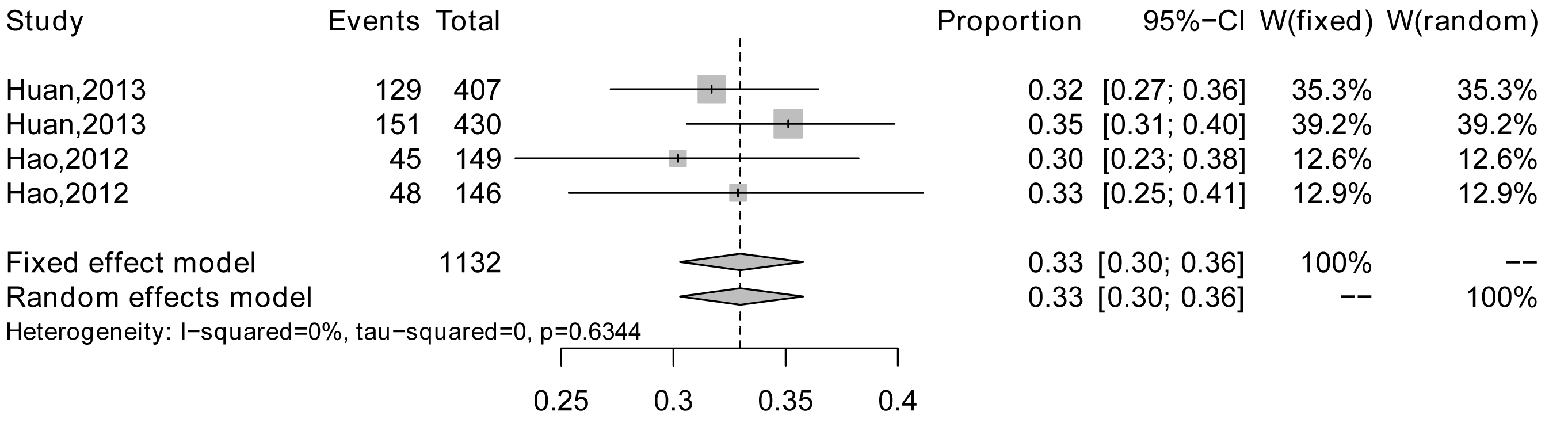

Supplement: Figure S4 — Forest plot of UAI prevalence with casual male partners among MSM in China. (TIF) [file pone.0098366.s004.tif]

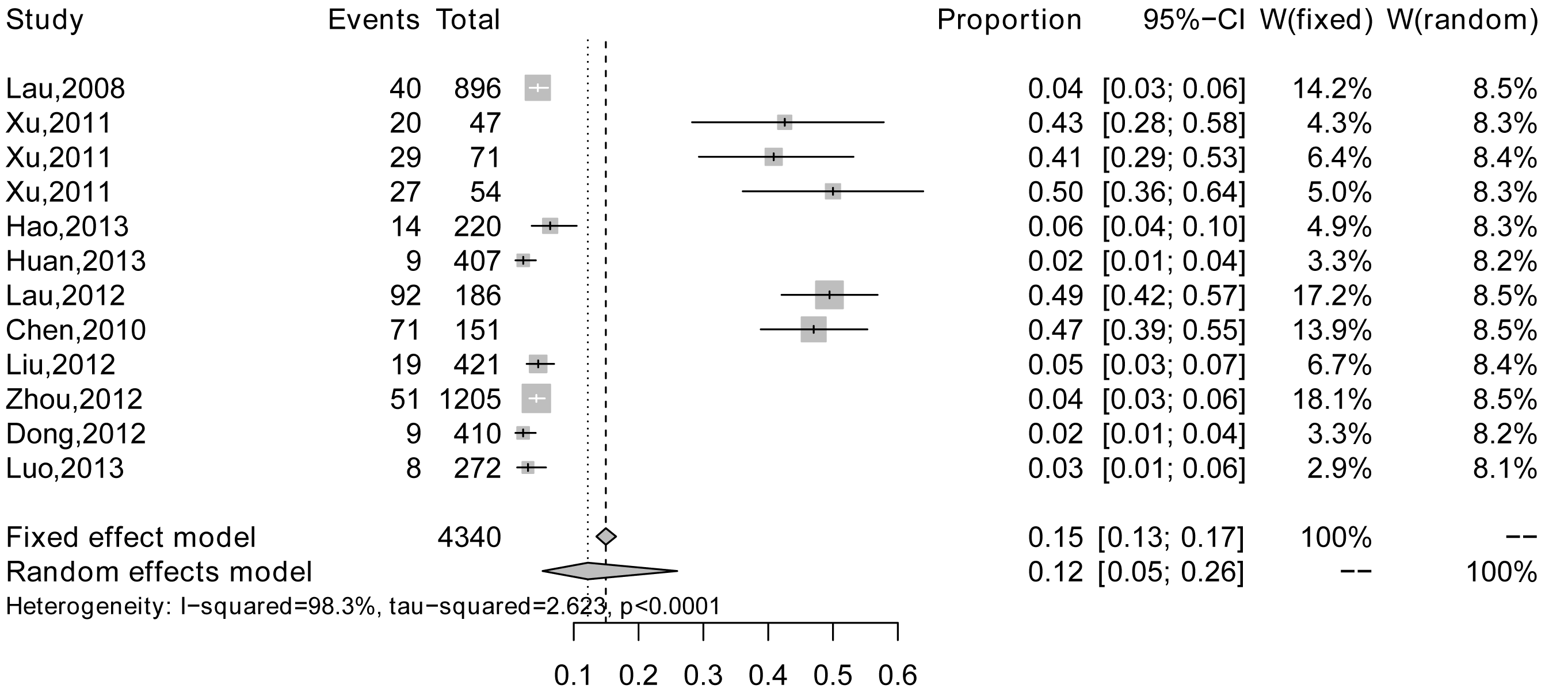

Supplement: Figure S5 — Forest plot of UAI prevalence with commercial male partners among MSM in China. (TIF) [file pone.0098366.s005.tif]

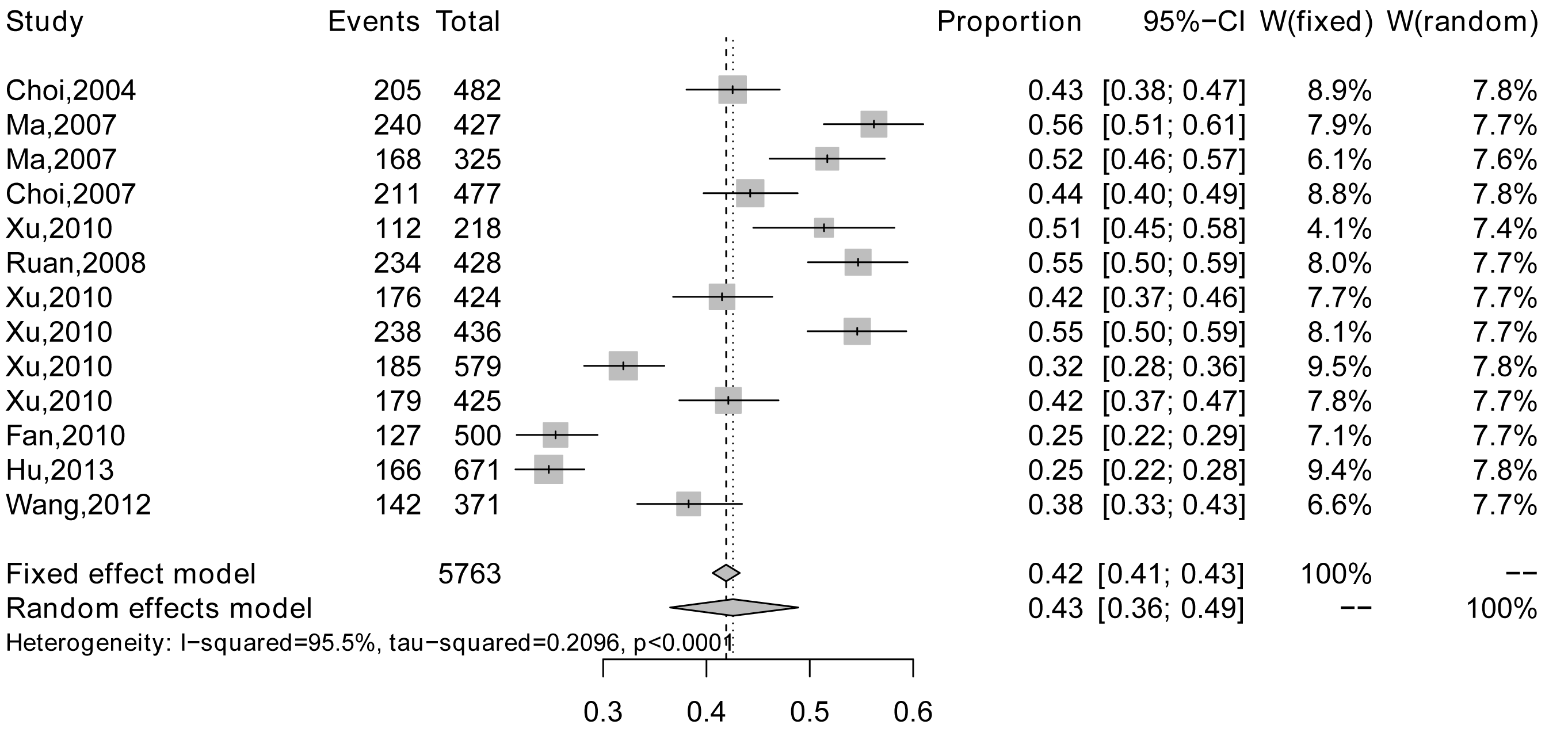

Supplement: Figure S6 — Forest plot of UIAI prevalence with any male partner among MSM in China. (TIF) [file pone.0098366.s006.tif]

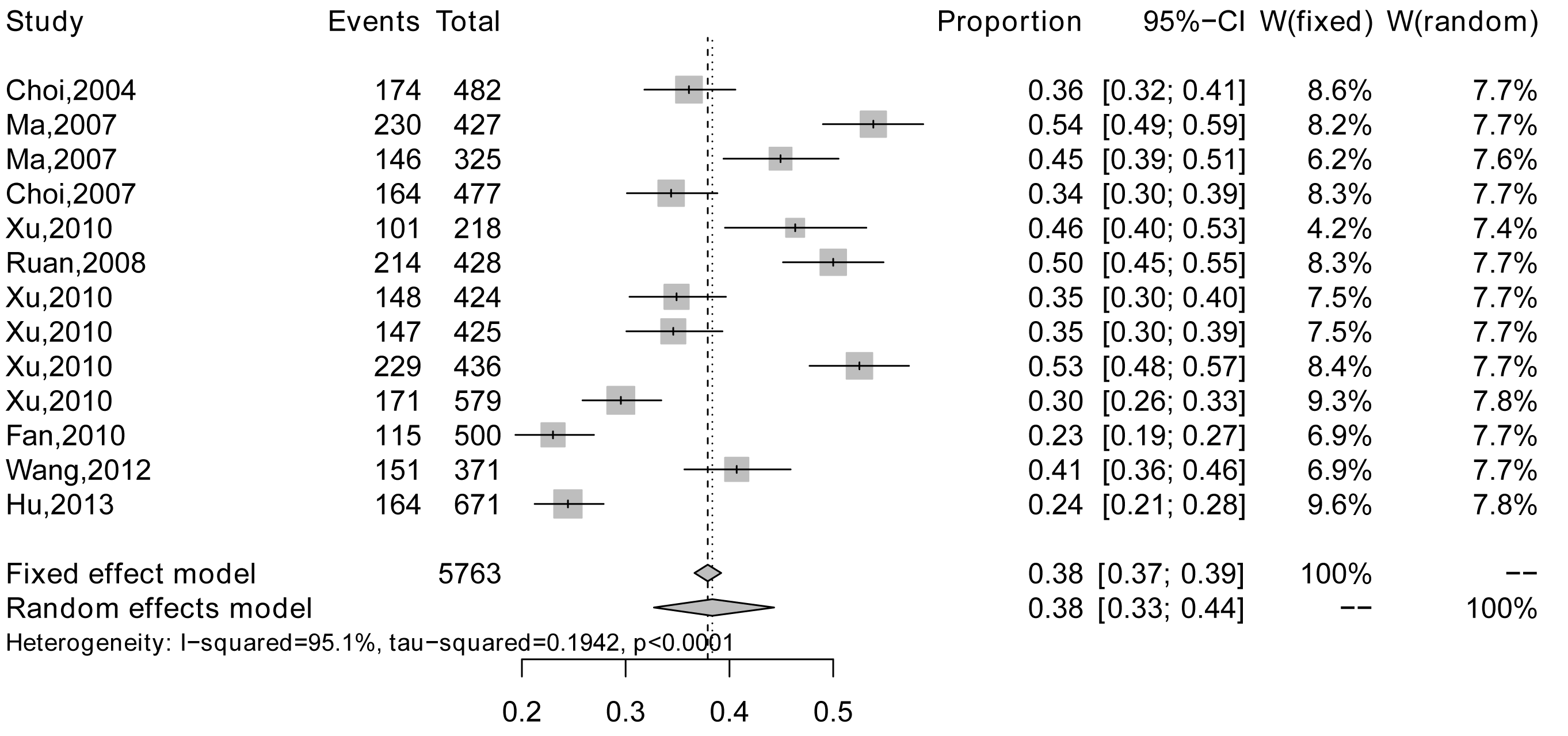

Supplement: Figure S7 — Forest plot of URAI prevalence with any male partner among MSM in China. (TIF) [file pone.0098366.s007.tif]

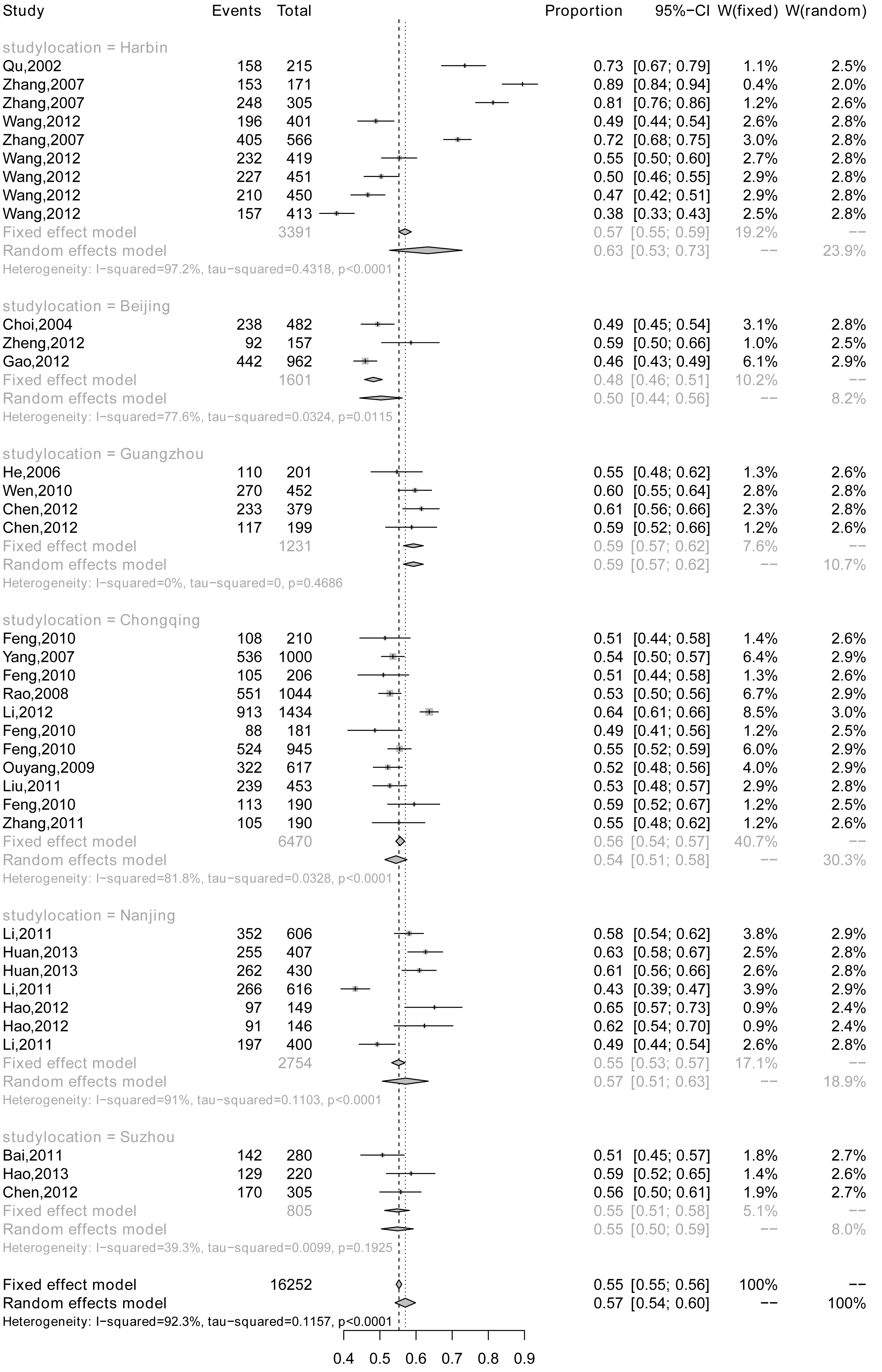

Supplement: Figure S8 — Subgroups analysis of UAI prevalence with any male partner among MSM in different cities of China. (TIF) [file pone.0098366.s008.tif]
