# Supplementary material for: Prevalence of Unprotected Anal Intercourse among Men Who Have Sex with Men in China: An Updated Meta-Analysis
Source: PLoS One. 2014 May 29;9(5):e98366. doi: 10.1371/journal.pone.0098366 (PMC4038612; doi:10.1371/journal.pone.0098366)
Supplement: Checklist S2 — Quality assessment of included individual studies. (DOC) [file pone.0098366.s010.doc]

QUALITY ASSESSMENT OF CONCLUDED INDIVIDUAL STUDIES Boyle MH (1998) Guidelines for evaluating prevalence studies. Evidence Based Mental Health 1: 37-39.
2  Chow EP, Iu KI, Fu X, Wilson DP, Zhang L (2012) HIV and sexually transmissible infections among money boys in China: a data synthesis and meta-analysis. PLoS One 7: e48025., 2

Ref. number: ___________________ First author, year: ___________________

ID 	Question for Evaluating Prevalence Studies 	Decision Criteria 	Score 	
Q1	Was the target population defined clearly? Was the population from which the sample was drawn clearly defined?	Yes: Background information, including: (i) study duration (e.g. yyyy/mm or yyyy) and (ii) recruitment locations (e.g. gay venues, internet, peer-referral, informant-referral) were identified clearly 	1 	
		No: Background information: neither (i) study duration or (ii) recruitment locations were not identified 
	0 	
Q2	Was probability sampling used to identify potential respondents?	Yes: Probability sampling (including: simple random, systematic, stratified, cluster, two-stage and multi-stage sampling)was adopted 	1 	
		No: Non-probability sampling (including: purposive, quota, convenience, snowball and response-driven sampling) was adopted 	0 	
Q3	Did the characteristics of respondents match the target population?	Yes: Inclusion/exclusion criteria in sample selection, including (i) male, (ii) self-reported ever had sex with men were indentified clearly 	1 	
		No: Inclusion/exclusion criteria in sample selection: neither (i) male or (ii) self-reported ever had sex with men were not identified 	0 	
Q4	Was the response rate adequate?	Yes: Response rate was ≥ 80% 	1 	
		No: Response rate was not reported or < 80% 	0 	
Q5	Were the data collection methods standardised?	Yes: Identical methods of assessment and data collection were used to all respondents 	1 	
		No: Methods of assessment and data collection to all respondents were not identical 	0 	
Q6	Were measures shown to be reliable?	Yes: (i) Survey instrument: Survey was test-retested, piloted, adopted/adapted from other study (with reference) or tested by Cronbach's alpha , (ii) STD's clinical tests (if having STD's tests): name(s) of screening/confirmation test was identified clearly 	1 	
		No: (i) Survey instrument: No information was identified in regards to the reliability of the instrument, (ii) STD's clinical tests (if having STD's tests): name of screening/confirmation test was not provided	0 	
Q7	Were measures shown to be valid?	Yes: (i) Survey instrument: The frequency of unprotected anal intercourse or condom use (including no use and consistent) with men were reported clearly in the past 6 months among MSM, (ii) STD's clinical tests (if having STD's tests): name(s) of screening/confirmation test was identified clearly	1 	
		No: (i) Survey instrument: The frequency of unprotected anal intercourse or condom use were not provided, (ii) STD's clinical tests (if having STD's tests): name of screening/confirmation test was not provided	0 	
Q8	Were the statistical methods appropriate?	Yes: Confident intervals or SD/variance were given for the rate of unprotected anal intercourse	1 	
		No: Only the prevalence rate was given (CI or SD was not reported) 	0 	
