# Supplementary material for: Prevalence of Unprotected Anal Intercourse among Men Who Have Sex with Men in China: An Updated Meta-Analysis
Source: PLoS One. 2014 May 29;9(5):e98366. doi: 10.1371/journal.pone.0098366 (PMC4038612; doi:10.1371/journal.pone.0098366)
Supplement: Table S1 — Characteristics of studies included in this meta-analysis. (DOCX) [file pone.0098366.s011.docx]

**TABLE S1. Characteristics of Included in This Meta-analysis.**

| Source | Data collection period | Study location | Age range | Sample source | Sample method | Study type | Data collection method | HIV prevalence (%) | Recruit settings |
| --- | --- | --- | --- | --- | --- | --- | --- | --- | --- |
| Choi,2004 [1] | 2001.12-2002.1 | Beijing | m=27 | venues, referral | convenience sampling | cross-sectional | interviewer | 3.1 [2] | NR |
| Ma,2007 [3] | 2004.10-2005.1 | Beijing | 18-60,m=28.4 | referral | RDS | cross-sectional | interviewer, self | 0.4 | NR |
| Ma,2007 [4] | 2005.9-12 | Beijing | 16-71,m=28.9 | prior respondents, VCT of Beijing CDC | RDS | cross-sectional | interviewer, self | 4.6 | Beijing CDC |
| Zheng,2012 [5] | 2007.9-12 | Beijing | 17-32,22.7±2.8 | internet | convenience sampling | cross-sectional | self | 2.5 | Jingcheng Hospital |
| Gao,2012 [6] | 2009.8-12 | Beijing | 18-71,M=27 | internet, referral | convenience sampling | cross-sectional | interviewer | 6.34 | Jingcheng hospital |
| Fan,2010 [7] | 2009.9-10 | Beijing | m=29 | internet, venues | RDS | cross-sectional | self | 8 | Beijing CDC |
| Hu,2013 [8] | 2010.1-2011.7 | Beijing | 18-61 | venues, internet, referral | convenience sampling | cross-sectional | interviewer | 31.6 | Xicheng CDC, Jingcheng Hospital |
| Xu,2010 [9] | 2008.3-7 | Beijing  Chengdu, Sichuan  Harbin, Heilongjiang  Zhengzhou, Henan | m=29.2 of 4 cities | NR | RDS  snowball sampling  RDS  snowball sampling | cross-sectional | interviewer | 6  11.1  4.5  5.5 | NR |
| Choi,2007 [10] | 2004.12-2005.5 | Shanghai | 18-56,m=28 | venues | snowball sampling | cross-sectional | interviewer | 1.47 [11] | Shanghai CDC |
| He,2012 [12] | 2010.6-12 | Shanghai | 21-68,36.29±10.09 | referral | snowball sampling | cross-sectional | interviewer | positive | NR |
| Wang,2012 [13] | 2009.6-9 | Mianyang，Sichuan | 18-69,24±8.28 | NR | RDS | cohort | interviewer | 11 | NR |
| Wang,2013 [14] | 2010.8-2011.1 | Mianyang, Sichuan | 18-75,28.2±10.5 | venues | snowball sampling | cross-sectional | interviewer | NR | NR |
| Wang,2013 [15] | 2011.4-10 | Mianyang, Sichuan | 15-72,26.1±9.2 | venues, referral | snowball sampling | cross-sectional | self | 12.5 | NR |
| Duan,2013 [16] | 2006-2008 | Mianyang, Sichuan  Yibin, Sichuan | NR | venues, referral | convenience sampling | Intervention  control | NR | NR | NR |
| Song,2013 [17] | 2010.7-2011.2 | Chengdu, Sichuan and Guangzhou, Guangzhou | 34.3±9.5 | referral | snowball sampling | cross-sectional | interviewer | 26.2 | NGO |
| Huan,2013[18] | 2008.5-7 | Nanjing, Jiangsu | 28.6±8.8 | venues, internet, referral | RDS | cross-sectional | interviewer | 4.7 | VCT clinic |
| Huan,2013 [19] | 2008 | Nanjing, Jiangsu | NR | internet, venues | RDS | cohort | interviewer | 4.7 | VCT clinic |
| Hao,2012 [20] | 2008.5-2009.2 | Nanjing, Jiangsu | 18-73,28.5±8.6 of all participants | internet, venues | RDS | RCT | interviewer | NR | VCT clinic |
| Wang,2013 [21] | 2009.5-2010.7 | Nanjing，Jiangsu | NR | venues, referral | convenience sampling | cross-sectional | interviewer | NR | NR |
| Li,2011[22] | 2008.5-11  2009.5-11  2010.5 | Nanjing, Jiangsu | 28.04±8.4  28.09±8.47  30.48±9.39 | NR | snowball sampling | cross-sectional | interviewer | 4  2.1  2.8 | VCT clinic |
| Hao,2013 [23] | 2008.5-8 | Suzhou, Jiangsu | NR | internet | convenience sampling | cross-sectional | interviewer | 8.2 | venues |
| Bai,2011 [24] | 2008.5 | Suzhou, Jiangsu | 28.1±8.8 | internet | Snowball sampling | cross-sectional | interviewer | 7.1 | NR |
| Liu,2012 [25] | 2010 | Zhenjiang，Jiangsu | 27.4±9 | referral | snowball sampling | cohort | interviewer | 7.7 | Zhenjiang CDC, venue |
| Tang,2009 [26] | 2008.5-7 | Nanjing, Yangzhou, Suzhou in Jiangsu | 33.16±9.92  30.58±9.82 | NR | RDS, snowball sampling | case  control | NR | positive  negative | NR |
| Yan,2010 [27] | 2008.5-2009.7 | 3 cities in Jiangsu | 18-78,29.89±9.6 | NR | snowball sampling | cross-sectional | interviewer | 7.1 | NR |
| Chen,2012 [28] | 2009.4-7 | Yangzhou, Jiangsu  Suzhou, Jiangsu | 35±10.7  27.5±7.2 | internet, referral | snowball sampling | cross-sectional | interviewer | 10  9.2 | VCT clinic |
| He,2006 [29] | 2004.10-11 | Guangzhou, Guangdong | 16-66,m=29.5 | media, internet, referral | convenience sampling | cross-sectional | interviewer | 0 | Guangdong CDC |
| Chen,2012 [30] | 2008.5-8 | Guangzhou, Guangdong | 18-51 [31] | internet, venues | RDS | cross-sectional | interviewer | 5.2 | VCT clinic [31] |
| Wen,2010 [32] | 2008.10-12 | Guangzhou, Guangdong | 18-64,28±7 | venues | snowball sampling | cross-sectional | interviewer | 4.9 | NR |
| Cheng,2010[33] | 2009.5-8 | Guangzhou, Guangdong | 17-54,M=24 | venues | convenience sampling | cross-sectional | interviewer | 11.3 | NR |
| Cheng,2012 [34] | 2009.11-12 | Guangzhou, Guangdong | 16-51,27±5.8 | VCT, community center | convenience sampling | cross-sectional | NR | 11.3 | Guangzhou CDC, NGO |
| Lau,2012 [35] | 2007.12-2008.2 | Shenzhen, Guangdong | 22.41±2.2 [36] | venues | convenience sampling | cross-sectional | interviewer | NR | NR |
| Zhao,2013 [37] | 2008.4-2009.12 | Shenzhen, Guangdong | 18-62,30±7.5 | venues | time-location-sampling method | cross-sectional | self | 7 | venues |
| Xu,2010 [38] | 2006.8-9 | Shenyang, Liaoning | 18-60,m=27 | referral | convenience sampling | cohort | interviewer | negative | NR |
| Liu,2012 [39] | 2009.6-10 | Fushun and Huludao, Liaoning | 19-72,32.5±11.1 | venues | convenience sampling | cross-sectional | interviewer | NR | NR |
| Zhou,2012 [40] | 2010 | Shenyang, Dalian, Ma'anshan in Liaoning | 15-71,27.27±8.29 | referral | snowball sampling | cross-sectional | interviewer | 4.7 | NR |
| Qu,2002 [41] | 2001.11-12 | Harbin, Heilongjiang | 16-67,M=29 | venues | convenience sampling | cross-sectional | interviewer | 1.31 [42] | venues |
| Zhang,2007[43] | 2002  2004  2006 | Harbin, Heilongjiang | 18-75,M=26  18-67,M=29  18-69,M=27 | venues | convenience sampling | cross-sectional | interviewer | 1.3  0. 9  2.2 | NR |
| Wang,2012 [44] | 2006  2007  2008  2009  2010 | Harbin, Heilongjiang | NR | venues | Snowball sampling | cross-sectional | interviewer | 1  2.9  3.5  5.1  7.5 | VCT clinic |
| Yan,2009 [45] | 2008.4-7 | 4 cities in Heilongjiang | 18-70,m=26 | NR | RDS, snowball sampling | cross-sectional | interviewer | 2.3 | NR |
| Ruan,2008 [46] | 2007.3-7 | Jinan, Shandong | NR | NR | RDS | cross-sectional | NR | 0.5 | NR |
| Dong,2013 [47] | 2011.4-7 | Yinchuan，Ningxia | 18-62,30.5±8.5,M=29 | internet, venues | snowball sampling | cross-sectional | NR | 3.8 | NR |
| Rao,2008 [48] | 2007.7-9 | Chongqing | 18-77,28.9±9.7 | NR | snowball sampling | cross-sectional | interviewer | NR | NR |
| Feng,2010 [49] | 2006  2007  2008  2009 | Chongqing | m=20.2  m=20.3  m=21.4  m=21.4 | NR | snowball sampling | cross-sectional | interviewer | 4.3  3.9  11  11.1 | NR |
| Feng,2010 [50] | 2008.2-6 | Chongqing | NR | internet, non-internet | snowball sampling | cross-sectional | interviewer | 15.8 | NR |
| Ouyang,2009 [51] | 2008.2-6 | Chongqing | NR | NR | RDS | cross-sectional | interviewer | 16.8 | STD clinic |
| Li,2011 [52] | 2006,2007.7-9 | Chongqing | m=27 | venues | snowball sampling | cross-sectional | interviewer | 11.2 | NR |
| Yang,2007 [53] | 2006.7-10 | Chongqing | 15-68,27.8±9.1 | venues, referral | snowball sampling | cross-sectional | interviewer | 10.4 | NR |
| Liu,2011 [54] | 2009.3-10 | Chongqing | 27.6±6.2 | internet, venues | snowball sampling | cohort | NR | negative | NR |
| Zhang,2011 [55] | 2009 | Chongqing | 16-47,24.2±6 | venues, referral | snowball sampling | cross-sectional | interviewer | 11.6 | NR |
| Xu,2011 [56] | 2006.4-7  2007.4-7  2008.4-7 | Chongqing | NR | NR | Snowball sampling | cross-sectional | interviewer | 12.8  9.9  7.7 | NR |
| Liu,2010 [57] | 2008.5-7 | Wuhan，Hubei | 18-60，28.27±8.43 [58] | referral | RDS | cross-sectional | interviewer, self | 6.6 | VCT clinic [58] |
| Zhang，2010 [59] | 2009 | Ningbo，Zhejiang | 18-67,28.1±7.8 | internet, venues | Snowball sampling | cross-sectional | interviewer, self | 4.2 | NR |
| Dong,2012 [60] | 2011.6-9 | Huzhou，Zhejiang | 15-47,25.5±4.5 | internet | convenience sampling | cross-sectional | self | NR | internet |
| Zhu,2008 [61] | 2006.6-12 | Hefei，Anhui | 20.7±2.3 | venues, internet, referral | RDS | cross-sectional | interviewer, self | NR | interviewer office, MSM home, site designated by MSM |
| Ma,2012 [62] | 2011.5-11 | Lu'an，Anhui | 18-52,m=27 | NR | snowball sampling | cross-sectional | self | NR | NR |
| Luo,2013 [63] | 2012.7-8 | Huainan，Anhui | 19-49,28±6 | venues | snowball sampling | cross-sectional | interviewer | NR | venues |
| Lau,2008 [64] | 2003.12-2004.2,2005.6-8,2006.4-5 | Kunming，Yunnan | 15-75 | venues, internet, referral | convenience sampling | cross-sectional | interviewer | NR | NR |
| Chow，2013 [65] | 2010.1-8  2011.1-8 | Yuxi，Yunnan | 33.3±11.3  33.1±11.1 | venues | Snowball sampling | cross-sectional | interviewer | 12.3  9.8 | NR |
| Wu,2013 [66] | 2011.3-9 | Tianjin | 17-76,36.3 | venue | convenience sampling | cross-sectional | interviewer | 16.6 | venue |
| Qu,2013 [67] | 2011.4-7 | Hohhot, Baotou in Inner Mongolia | 18-68,32.44±9.34 | venues | convenience sampling | cross-sectional | interviewer | 21.32 | venues |
| Zheng,2013 [68] | 2011.4-7 | Nanning, Liuzhou, Guilin in Guangxi | 15-73 | internet, venues | convenience sampling | cross-sectional | interviewer | 4.6 | NR |

Note: 1. A small number of information are from duplicate articles.

2.NR represents no reported in the studies.

3.m means mean of age, M means median of age.

4.RDS means Respondent-Driven Sampling; NGO means Non-Governmental Organization.

**REFERENCES**

1. Choi KH, Gibson DR, Han L, Guo Y (2004) High levels of unprotected sex with men and women among men who have sex with men: a potential bridge of HIV transmission in Beijing, China. AIDS Educ Prev 16: 19-30.

2. Choi KH, Liu H, Guo Y, Han L, Mandel JS, et al. (2003) Emerging HIV-1 epidemic in China in men who have sex with men. Lancet 361: 2125-2126.

3. Ma XY, Zhang QY, Zhao JK, He X, Du H, et al. (2007) Feasibility of using respondent driven sampling in HIV/STD epidemiological survey among MSM. Chin J AIDS STD 13: 311-313.

4. Ma XY, Zhang QY, He X, Zhao JK, Li Y, et al. (2007) Epidemiological study on the status of HIV/STD and relative behaviors among MSM in Beijing. Chin J Epidemiol 28: 851-855.

5. Zheng J, Wu Z, Poundstone KE, Pang L, Rou K (2012) HIV, syphilis infection, and risky sexual behaviors among male university students who have sex with men in Beijing, China: a cross-sectional study. AIDS Educ Prev 24: 78-88.

6. Gao YJ, Yu MR, Li SM, Zhang Z, Li DL, et al. (2012) Prevalence and predictors of HIV, syphilis and herpes simplex type 2 virus (HSV-2) infections among the men who have sex with men (MSM) in Beijing. Chin J Public Health 28: 451-453.

7. Fan S, Lu H, Ma X, Sun Y, He X, et al. (2012) Behavioral and serologic survey of men who have sex with men in Beijing, China: implication for HIV intervention. AIDS Patient Care STDs 26: 148-155.

8. Hu Y, Qian HZ, Sun J, Gao L, Yin L, et al. (2013) Anal human papillomavirus infection among HIV-infected and uninfected men who have sex with men in Beijing, China. J Acquir Immune Defic Syndr 64: 103-114.

9. Xu J, Han DL, Liu Z, Ma XY, Wang LL, et al. (2010) The prevalence of HIV infection and the risk factors among MSM in 4 cities, China. Chin J Prev Med 44: 975-980.

10. Choi KH, Ning Z, Gregorich SE, Pan QC (2007) The influence of social and sexual networks in the spread of HIV and syphilis among men who have sex with men in Shanghai, China. J Acquir Immune Defic Syndr 45: 77-84.

11. Choi KH, Hudes ES, Steward WT (2008) Social discrimination, concurrent sexual partnerships, and HIV risk among men who have sex with men in Shanghai, China. AIDS Behav 12: S71-77.

12. He H, Wang M, Zhang HB, Song DD, She M, et al. (2012) The unprotected sexal behaviors and its influencing factors among HIV-infected men who have sex with men in Shanghai, China. Chin J Prev Med 46: 976-981.

13. Wang Y, Xu J, Li ZJ, Zhang GG, Li LL, et al. (2012) Analysis of HIV/syphilis infection among MSM seeking sex partners in different venues and related behavioral features. Chin J AIDS STD 18: 180-183.

14. Wang Y, Li LL, Zhang GG, Fan J, Zhao XH, et al. (2013) Research on the network activities of MSM and characteristics of the related social behavior of the network sexual partners. Practical Preventive Medicine 20: 260-263.

15. Wang Y, Li LL, Fan J, Zhao XH, Yang XL, et al. (2013) Analysis on relationship of drug use behavior and HIV infection and related behaviors among men who have sex with men. Chin J Behav Med & Brain Sci 22: 364-366.

16. Duan Y, Zhang H, Wang J, Wei S, Yu F, et al. (2013) Community-based peer intervention to reduce HIV risk among men who have sex with men in Sichuan province, China. AIDS Educ Prev 25: 38-48.

17. Song D, Zhang H, Wang J, Liu Q, Wang X, et al. (2013) Prevalence and correlates of HIV infection and unrecognized HIV status among men who have sex with men and women in Chengdu and Guangzhou, China. AIDS Behav 17: 2395-2404.

18. Huan X, Hao C, Yan H, Guan W, Xu X, et al. (2013) High prevalence of HIV and syphilis among men who have sex with men recruited by respondent-driven Sasing in a city in eastern China. Asia Pac J Public Health [Epub ahead of print].

19. Huan X, Tang W, Babu GR, Li J, Zhang M, et al. (2013) HIV risk-reduction counseling and testing on behavior change of MSM. PLoS One 8: e69740.

20. Hao C, Huan X, Yan H, Yang H, Guan W, et al. (2012) A randomized controlled trial to evaluate the relative efficacy of enhanced versus standard voluntary counseling and testing on promoting condom use among men who have sex with men in China. AIDS Behav 16: 1138-1147.

21. Wang Z, Lau JT, Hao C, Yang H, Huan X, et al. (2013) Syphilis-related perceptions not associated with risk behaviors among men who have sex with men having regular male sex partner(s) in Nanjing, China. AIDS Care 25: 1010-1017.

22. Li JJ, Huan XP, Yan HJ, Zhang M, Tang WM, et al. (2011) Effectiveness of behavioral intervention combined with voluntary counseling and testing on high risk behaviors among MSM. Chin Prev Med 12: 666-669.

23. Hao C, Lau JT, Zhao X, Yang H, Huan X, et al. (2014) Associations between perceived characteristics of the peer social network involving significant others and risk of HIV transmission among men who have sex with men in China. AIDS Behav 18: 99-110.

24. Bai H, Huan X, Tang W, Chen X, Yan H, et al. (2011) A survey of HIV infection and related high-risk factors among men who have sex with men in Suzhou, Jiangsu, China. J Biomed Res 25: 17-24.

25. Liu XX, Zhang MH, Chen X, Zhu YY, Zhu Q, et al. (2012) Analysis on the seroconversion of HIV/syphilis and the predictors of cohort retention among men who have sex with men in Zhenjiang: a prospective cohort study. Chin J Dis Control Prev 16: 1024-1027.

26. Tang WM, Yan HJ, Liu XY, Wang N, Zhang M, et al. (2009) Factors associated with HIV infection among men who have sex with men in Nanjing, Suzhou and Yangzhou: a 1:4 matched case-control study. Chin J Epidemiol 30: 448-451.

27. Yan HJ, Zhang M, Li JJ, Guan WH, Hu HY, et al. (2010) Prevelence of sexually transmitted infections and the behavior characteristics of men who have sex with men in Jiangsu Province. Chin Prev Med 11: 1249-1252.

28. Chen X, Zhu YY, Huan XP, Wang Q, Zheng H, et al. (2012) Comparison of HIV/AIDS prevalence among men who have sex with men between Suzhou and Yangzhou city. Chin J Public Health 28: 1549-1552.

29. He Q, Wang Y, Lin P, Liu Y, Yang F, et al. (2006) Potential bridges for HIV infection to men who have sex with men in Guangzhou, China. AIDS Behav 10: S17-23.

30. Chen AD, Zhong F, Fan LR, Wen F, Cheng WB, et al. (2012) Unprotected anal intercourse and related factors among men who have sex with men in Guangzhou City. South China J Prev Med 38: 1-6.

31. Zhong F, Lin P, Xu H, Wang Y, Wang M, et al. (2011) Possible increase in HIV and syphilis prevalence among men who have sex with men in Guangzhou, China: results from a respondent-driven sampling survey. AIDS Behav 15: 1058-1066.

32. Wen F, Zhong F, Cheng WB, Gao K, Luo BL, et al. (2010) HIV and current syphilis prevalence and related factors among men who have sex with men in Guangzhou. South China J Prev Med 36: 19-23.

33. Cheng WB, Zhong F, Wen F, Gao K, Liu JW, et al. (2010) Investigation of HIV and syphilis infection and AIDS-related behaviors among money boys, in Guangzhou, China. Chin J Prev Med 44: 1027-1031.

34. Cheng WB, Zhong F, Wen F, Xu HF (2012) Effects on AIDS-related knowledge and attitudes on high risk sexual behavior among men who have sex with men. Chin J Dis Control Prev 16: 1064-1067.

35. Lau JT, Cai W, Tsui HY, Chen L, Cheng J, et al. (2012) Unprotected anal intercourse behavior and intention among male sex workers in Shenzhen serving cross-boundary male clients coming from Hong Kong, China - prevalence and associated factors. AIDS Care 24: 59-70.

36. Lau JT, Cai WD, Tsui HY, Chen L, Cheng JQ (2009) Psychosocial factors in association with condom use during commercial sex among migrant male sex workers living in Shenzhen, mainland China who serve cross-border Hong Kong male clients. AIDS Behav 13: 939-948.

37. Zhao J, Chen L, Cai WD, Tan JG, Tan W, et al. (2013) HIV infection and sexual behaviors among non-commercial men who have sex with men at different venues. Arch Sex Behav [Epub ahead of print].

38. Xu JJ, Zhang M, Brown K, Reilly K, Wang H, et al. (2010) Syphilis and HIV seroconversion among a 12-month prospective cohort of men who have sex with men in Shenyang, China. Sex Transm Dis 37: 432-439.

39. Liu J, Qu B, Ezeakile MC, Zhang Y (2012) Factors associated with unprotected anal intercourse among men who have sex with men in Liaoning Province, China. PLoS One 7: e50493.

40. Zhou JL, Zhang Y, Shi JP, Wang L (2012) Knowledge and behavior about HIV/AIDS and HIV infection among MSM in Liaoning province. Chin J Public Health 28: 806-808.

41. Qu SQ, Zhang DP, Wu YH, Zhu H, Cui Y, et al. (2002) A survey of knowledgy, attitude and practice related to HIV/AIDS among men who have sex with men in a northeastern city of China. Chin J AIDS STD 8: 338-340, 370.

42. Qu SQ, Zhang DP, Zhu H, Zheng XW, Wu YH (2002) Seroprevalence of HIV and risk behaviors among men who have sex with men in a northeast city of China. Chin J AIDS STD: 145-147, 161.

43. Zhang D, Bi P, Lv F, Zhang J, Hiller JE (2007) Changes in HIV prevalence and sexual behavior among men who have sex with men in a northern Chinese city: 2002-2006. J Infect 55: 456-463.

44. Wang K, Yan H, Liu Y, Leng Z, Wang B, et al. (2012) Increasing prevalence of HIV and syphilis but decreasing rate of self-reported unprotected anal intercourse among men who had sex with men in Harbin, China: results of five consecutive surveys from 2006 to 2010. Int J Epidemiol 41: 423-432.

45. Yan HM, Wang KL, ZHAO DH, Hui S, Luo GL, et al. (2009) An analysis of HIV/AIDS related high-risk behavior, infection status and impact factors among MSM in Heilongjiang Province. Chin J AIDS STD 15: 288-291.

46. Ruan S, Yang H, Zhu Y, Ma Y, Li J, et al. (2008) HIV prevalence and correlates of unprotected anal intercourse among men who have sex with men, Jinan, China. AIDS Behav 12: 469-475.

47. Dong XF, Lei J, Miao ZF, Han X, Yang ZG, et al. (2013) A research of the influencing factors of AIDS high-risky behavior among 400 MSM. Journal of Ningxia Medical University 35: 277-279.

48. Rao XM, Xiong HY, Ding XB, Feng LG (2008) Study on status of unprotected anal sex and its associated factors among men who have sex with men in Chongqing. Chongqing Medicine 37: 2335-2337.

49. Feng LG, Ding XB, Lu RR, Xu SM, Guo XJ, et al. (2010) Trend on prevalence of HIV and syphilis and estimated HIV incidence among young male students who have sex with men. Acta Academiae Medicinae Militaris Tertiate 32: 2644-2646.

50. Feng LG, Ding XB, Xu J, Ouyang L, Xu SM, et al. (2010) Study on HIV syphilis and HCV prevalence and its associated factors among internet MSM comparison to non-internet MSM in Chongqing. J Trop Med 10: 78-82.

51. Ouyang L, Feng LG, Ding XB, Zhao JK, Xu J, et al. (2009) A respondent-driven sampling survey on HIV and risk factors among men who have sex with men in Chongqing. Chin J Epidemiol 30: 1001-1004.

52. Li CM, Jia YJ, Liu JB, Ding XB, Xiao Y (2011) Prevalence and predictors of unprotected anal sex among men who have sex with men in Chongqing municipality, China. Chin J Public Health 27: 351-352.

53. Yang X, Yi D, Ding XB (2007) High risk behaviors of HIV/AIDS in male-to-male sex and its influencing factors in 1000 men in a city. Acad J Sec Mil Med Univ 28: 1223-1228.

54. Liu ZY, Wu GH, Xu J, Ding XB, Mi GD, et al. (2011) Factors associated with cohort retention among men who have sex with men. Chin J AIDS STD 17: 646-650.

55. Zhang QP, Ding XB, Feng LG, Xu J, Lu RR, et al. (2011) The prevalence of HIV and syphilis and its influencing factors among male sex workers. ACTA Academiae Medicinae Militaris Tertiate 33: 318-321.

56. Xu J, He J, Feng LG, Ding XB, Xu SM, et al. (2011) Prevalence of HIV and syphilis and its risk behavior among money boys in Chongqing (2006-2008). J Trop Med 11: 1199-1202.

57. Liu PL, Yao ZZ, Shi WD, Ding J, Li SL, et al. (2010) Epidemiological study on the status of HIV/STD among MSM in Wuhan City. Chin J Dis Control Prev 14: 917-919.

58. Shi WD, Li G, Yang T, Zhou W, Liu PL, et al. (2009) Survey of High Risk Sexual Behaviors and HIV ,Syphilis, HCV among 456 Male Homosexuals in Wuhan. Medical and Society 22: 42-43.

59. Zhang DD, Zhang Y, Li HL, Wang JX, Zhang Z, et al. (2010) Investigation of HIV, syphilis and HCV infection and sexual behavioral characteristics among MSM in Ningbo City. Zhejiang Preventive Medicine 22: 1-3.

60. Dong ZQ, Jin MH, Qiu ZH, Yang ZR, Zhang SC, et al. (2012) Survey of AIDS related knowledge awareness and high-risk behavior in 410 men who have sex with men in Huzhou，Zhejiang. Disease Surveillance 27: 623-626.

61. Zhu JL, Zhang HB, Zheng YZ, Choi KH, Zhu YB (2008) Association between HIV-risk behaviors of young men who have sex with men and characteristics of their core social networks. Chin J Public Health 24: 400-402.

62. Ma GY, Li KC, Chen HY, Chen J, Xu PP (2012) Investigation of HIV, syphilis and HCV infection and sexual behavioral characteristics among MSM in Ningbo City. Anhui J Prev Med 18: 332-335.

63. Luo Y, Bao QG, Zhang XH (2013) Survey on the AIDS-related high risk behaviors and associated factors of men who have sex with men in Huainan city. Anhui J Prev Med 19: 83-86, 93.

64. Lau JT, Wang M, Wong HN, Tsui HY, Jia M, et al. (2008) Prevalence of bisexual behaviors among men who have sex with men (MSM) in China and associations between condom use in MSM and heterosexual behaviors. Sex Transm Dis 35: 406-413.

65. Chow EP, Chen L, Jing J, Gao L, Zhang J, et al. (2013) HIV disease burden and related risk behaviours among men who have sex with men in Yuxi Prefecture, Yunnan Province, China: 2010-2011. AIDS Behav 17: 2387-2394.

66. Wu D, Chen SH, Yang J, Yu MH, Gao YJ, et al. (2013) HIV prevalence and associated factors among gay bathhouse attendees in China. Chin J AIDS STD 19: 127-130.

67. Qu L, Gao YM, Qing H, Yang JY, Tao B (2013) Study on HIV infection and behavior among MSM in different venues. Practical Preventive Medicine 20: 145-149.

68. Zheng WB, Zhu QY, Lan GH, Zhu JH, Dong BQ (2013) Efficacy of AIDS intervention service among MSM in Guangxi and its influential factors. China Tropical Medicine 13: 175-179.
